# Dietary Fat and Protein Intake and Their Impact on Glycemic Control in Pediatric Type 1 Diabetes: A Narrative Review

**DOI:** 10.3390/children12121664

**Published:** 2025-12-08

**Authors:** Kosmas Margaritis, Vasiliki Rengina Tsinopoulou, Eleni P. Kotanidou, Assimina Galli-Tsinopoulou

**Affiliations:** Unit of Pediatric Endocrinology, Diabetes and Metabolism—Collaborative Sweet Center, 2nd Department of Paediatrics, School of Medicine, Faculty of Health Sciences, Aristotle University of Thessaloniki, AHEPA University General Hospital, Stilponos Kyriakidi 1, 54636 Thessaloniki, Greece; kosmas.d.margaritis@gmail.com (K.M.); vasotsino@gmail.com (V.R.T.); epkotanidou@auth.gr (E.P.K.)

**Keywords:** type 1 diabetes, children, adolescents, protein, fat, insulin dosing, macronutrients, glycemic control

## Abstract

**Highlights:**

**What are the main findings?**

- Dietary protein and fat significantly alter postprandial glucose levels in children and adolescents with type 1 diabetes, leading to delayed and prolonged postprandial hyperglycemia.
- Insulin dose adjustments should be made in the form of dual wave boluses or extended insulin boluses, in order to maintain or even improve glycemic control.

**What is the implication of the main finding?**

- Higher protein and fat dietary intake is more likely to disrupt glycemic control rather than enhance it, and personalization of insulin dose strategies should be performed.
- A balanced macronutrient distribution should be advised, based on ISPAD (2024) and ADA (2025) recommendations, in order to promote growth and cardiovascular health.

**Abstract:**

Carbohydrates have been the center of type 1 diabetes dietary management. Emerging evidence highlights the important effects of fat and protein in postprandial hyperglycemia, suggesting that an increase in daily fat and protein intake, combined with appropriate insulin dose adjustments, might lead to better glycemic control. It is well studied that meals containing fat or protein lead to late postprandial hyperglycemia. Studies that researched the use of these macronutrients observed the need for extended or dual wave boluses to achieve euglycemia and that no consistent improvement in HbA1c or time in range was related to higher protein or fat intake. Optimizing glycemic control in pediatric T1D requires strategies beyond carbohydrate counting. While balanced macronutrient distribution remains the main solid factor in stable glycemic profiles, more studies regarding the variety of macronutrients’ formulation in optimizing glycemic control are needed.

## 1. Introduction

Nutrition has been a fundamental element of diabetes management, and education regarding carbohydrate estimation is the main component of optimizing glycemic control in patients with type 1 diabetes. Carbohydrate calculation is the preferred option of meal planning methods in children and adolescents with type 1 diabetes mellitus (T1DM), as it provides better glycemic control and flexibility. However, the daily dietary intake of a child/adolescent does not consist solely of carbohydrates and, furthermore, does not always align with the recommended nutritional suggestions, as stated by ISPAD. In that context, fat and protein are also major components of their diet, and their delayed effect on postprandial hyperglycemia is often overlooked and underestimated. Dietary fat delays gastric emptying, reduces insulin sensitivity, and increases hepatic glucose production, whereas protein intake causes endogenous glucose production via an increase in circulating amino acids, resulting in delayed, elevated postprandial glucose levels, appearing 3–5 h after a meal [1,2]. As is already known, patients with type 1 diabetes have a total deficiency of insulin secretion due to the destruction of pancreatic beta cells and are in need of exogenous administration of insulin via basal and prandial doses. The amount of carbohydrates consumed has been referred to as the main regulator of glycemic response and the following prandial insulin dose, while the effect of fat and protein is usually misjudged. In fact, few adjustments to insulin dosing are made when dietary fat and protein are taken into consideration, a practice that leaves the child/adolescent exposed to late hyperglycemia, and therefore leads to suboptimal glycemic control and glycemic variability [3]. The most recent clinical practice consensus from ISPAD states that fat and protein should be taken into consideration when mixed meals are ingested. Furthermore, there is a trend towards high-energy dietary patterns derived from fat and protein and a decrease in carbohydrate intake. The limited data regarding this subject report mixed findings, with higher protein intake being associated with better and more stable glycemia, while higher fat intake may contribute to delayed postprandial hypoglycemia [4,5]. At the same time, ISPAD underscores the importance of balanced macronutrient intake to support normal growth and to avoid potential long-term cardiometabolic harm that might accompany routine promotion of very-high-fat or very-low-carbohydrate diets in youth [2]. Experimental meal studies show that high-fat or high-protein meals lead to early peaks in glucose but a markedly more pronounced glucose decrease several hours after eating. A very interesting fact is that when fat and protein are combined in a meal, the effect is additive [1,6]. Importantly, studies in adolescents using insulin pumps have shown that adjusting insulin delivery for protein and fat can reduce late postprandial hyperglycemia compared to carbohydrate counting alone [7,8,9]. These interventions, however, have not been tested in large-scale T1DM cohorts, as well as in multiple-daily injection patients, and therefore leave important questions open about generalizability. This narrative review aims to present the different mechanisms in which carbohydrate, protein, and fat influence postprandial hyperglycemia, reviewing recent published knowledge on the glycemic impact of high-fat and high-protein meals, as well as the required insulin doses and adjustments. This review also aims to provide clinicians and researchers with a coherent framework for addressing the problem of late postprandial hyperglycemia. By integrating the current literature with ISPAD’s guidance and highlighting clinical implications, the goal is to move practice beyond carbohydrate counting while safeguarding the unique nutritional needs of growing patients.

## 2. Materials and Methods

This narrative review was conducted to synthesize current knowledge regarding the effects of dietary protein and fat on postprandial glucose levels, and to further examine whether the increase in these macronutrients in the daily intake of children and adolescents with type 1 diabetes leads to optimal glycemic management. A literature search was performed in PubMed, Scopus, and Web of Science through October 2025, using combinations of the following keywords: “type 1 diabetes,” “children,” “adolescents,” “protein,” “fat,” “postprandial,” “insulin dosing,” “macronutrients,” and “glycemic control”. The ISPAD Clinical Practice Consensus Guidelines (2018 and 2024 updates), as well as the American Diabetes Association Standard of Care 2025, were included to contextualize emerging evidence. A high-fat diet was defined as ≥30–35 g per meal or >35% of total energy, while a high-protein diet was defined as ≥20–25 g per meal or >20% of total energy, as stated by ISPAD and ADA. Articles on type 2 diabetes patients, focusing solely on adults or animal models, addressing exercise-only interventions, or on carbohydrate counting without fat/protein evaluation were excluded, as were studies in languages other than English. Screening of searching results was performed by two of the authors (K.M. and V.R.T.), and the full text of all relevant studies was reviewed. In case of discrepancy, a third author (E.P.K.) contributed to the final selection.

## 3. Results/Discussion

### 3.1. Physiological Mechanisms of Fat and Protein in Glycemia

The predominant macronutrients that affect postprandial glucose levels are carbohydrates, and when ingested, they are metabolized into glucose and other monosaccharides and transported into the bloodstream, therefore causing a direct increase in glucose concentration [10]. However, recent data has stated that dietary fat and protein also play a substantial role in postprandial glycemic profile. Fat, due to not being a sole dietary element, does not alter the glycemic response, although it affects it by modifying the carbohydrate-induced glycemic response. The addition of fat in a meal containing carbohydrates causes a reduction in the rise in glucose levels 1–3 h postprandially, as well as the prolongation of these less elevated levels [10,11]. This is better shown in studies comparing the glucose response that followed meals containing high fat and meals containing a combination of high carbohydrates and low fat. The latter meal caused higher glucose levels and a less extended glycemic response [12]. Furthermore, studies that examined data from CGMs reported that fat leads to late postprandial hyperglycemia (>3 h), a factor surely affecting the glycemic control and management. The mechanisms involved include increased gluconeogenesis, in which the hydrolization of triacylglycerol (the main lipid in the human diet) leads to glycerol and, via pyruvate, the synthesis of more glucose [13]. Another factor involved is the direct effect of free fatty acids on insulin secretion through interaction with membrane receptors of beta cells, as well as the secretion of other glucose metabolism-oriented hormones (glucose, Glp-1, GIH, ghrelin). For example, studies have shown that meals with high carbohydrates and fat cause an increase in ghrelin, GLP-1, and GIP. The delayed gastric emptying is another major factor affecting the glucose response. A meal containing fat delays transportation from the stomach to the duodenum and therefore causes a reduction in the absorption of glucose. The glycemic and insulin response is delayed in healthy children and adolescents, and when the phenomenon of delayed gastric emptying is accompanied by insulin doses in type 1 diabetes patients, hypoglycemia events are recorded. Dietary protein, on the other hand, has no immediate effect in healthy individuals on postprandial glucose levels. A meal containing protein, however, provides the ingredients for gluconeogenesis and, therefore, a delayed modest increase in blood glucose levels (3–5 h) [14,15,16,17]. In healthy individuals a subsequent signaling of insulin and glucagon secretion helps maintain the balance in glucose levels, but in type 1 diabetes patients, it leads to hyperglycemia. Furthermore, high protein meals tend to alter the secretion of hormones that are involved in glucose homeostasis, such as glucagon, which was already mentioned, and cortisol, which causes higher insulin requirements and resistance. Other hormones that are affected include growth hormone, IGF-1, and ghrelin. Last but not least, a meal that contains both fat and protein is reported to cause acute insulin resistance, as it increases the hepatic glucose output and subsequently has a glycemic impact that could be characterized as additive [18,19,20,21].

### 3.2. Dietary Trends in Macronutrient Intake

The cornerstone of type 1 diabetes mellitus management is the patient’s diet and macronutrient intake. The main focus has been to provide each patient with a balanced dietary plan, composed of carbohydrates, protein, and fat, taking into consideration, besides the need for insulin treatment, the patient’s growth and overall development. Total energy distribution reflects a balanced macronutrient approach. Historically, this balance was shifted towards carbohydrate intake, which was the main focus of insulin therapy management, with dietary recommendations emphasizing carbohydrate counting as the primary strategy for glycemic control. Over the past decade, as many studies have shed light on the effect of protein and fat on glucose levels, the rise in flexible insulin dosing strategies, as well as public perceptions of carbohydrates, has shifted the approach in dietary management considerably, paralleling broader shifts toward lower carbohydrate and higher fat or protein intake. There is a relatively significant increase in “moderate- to low-carbohydrate” diet adoption, with an emphasis on high protein and high fat intake [4,5,22]. Advances in continuous glucose monitoring (CGM) have made postprandial glucose excursions more visible, reinforcing the perception that carbohydrate restriction leads to tighter control. In addition, flexibility in insulin therapy, particularly with hybrid closed-loop systems, has allowed greater dietary experimentation [1]. Observational data from international cohorts indicate that many children with T1D now exceed recommended fat intakes (>35% of energy), with saturated fat frequently above guideline limits. The recent ISPAD 2024 Clinical Practice Consensus Guidelines, however, suggest 45–55% of total energy from carbohydrates, 15–20% from protein, and less than 35% from fat [2]. While these trends reflect attempts to improve glycemic control, current evidence suggests that higher fat and protein intakes do not consistently yield better metabolic outcomes and may introduce new management challenges. Overall, the literature suggests that while fat and protein are essential components of a healthy diet, increasing their proportion beyond ISPAD recommendations does not confer superior glycemic outcomes [1,2]. Instead, a balanced macronutrient intake—combined with individualized insulin adjustments that account for meal composition—appears most effective for achieving stable glucose control and supporting normal growth and development [23]. The apparent rise in fat and protein intake among pediatric T1D populations underscores the need for renewed emphasis on dietary education, ongoing nutrition monitoring, and long-term studies assessing the metabolic and cardiovascular implications of these shifts.

### 3.3. Effects of Fat and Protein Intake on Glycemic Control

As carbohydrate count has been one of the cornerstones of type 1 diabetes mellitus management for many years, interest has been shifted towards other macronutrients, with fat and protein being at the center of recent research studies. Improving glucose stability and reducing variability, and therefore obtaining better glycemic control, is the primary goal of diabetes management, and many studies have examined the way in which higher fat and protein intakes influence postprandial glucose excursions.

The metabolic impact of dietary fat and protein on postprandial glycemia has been explored through studies in children with type 1 diabetes mellitus that demonstrate that both macronutrients independently and additively modify glucose excursions. This effect is typically delayed and prolongs postprandial hyperglycemia compared with carbohydrate-only meals [11,12,13,24]. Van der Hoogt et al. examined the effects of high-fat, high-protein meals (HF/HP) on children and adolescents on insulin pump therapy. Higher concentrations of fat and protein in meals required significantly more total insulin, but also increased the time spent above the target glucose level. The amount of insulin needed for post-meal correction was up to eight times greater when large amounts of protein and fat were added to a carbohydrate meal. Furthermore, the duration of postprandial hyperglycemia increased [25]. Abdou et al. researched the postprandial levels of glucose after standard, HF, and HP meals. The results are inconsistent with those of other similar studies, with minimal differences in the time of peak hyperglycemia and its duration. High-protein meals are followed by delayed postprandial hyperglycemia, while high-fat meals are followed by earlier peak hyperglycemia—up to 2 h. While the etiology of these findings is based on the aforementioned pathophysiological mechanisms of fat and protein affecting glycemia, the study results indicate that the addition of these macronutrients to meals leads to various timeframes of hyperglycemia and should be taken into consideration when calculating the insulin boluses [26]. Smart et al. compared the effect of protein and fat on glucose levels and insulin requirements based on different quantities of fat and protein meals (LF/LP, LF/HP, HP/LF, HP/HF). When a more than usual amount of either protein or fat was added to the consumed meal, increased glucose excursions were reported, with the variable differing on each occasion being the time of peak hyperglycemia. The importance of this study is that it demonstrates the additive impact of protein and fat, because the greater increase in postprandial glucose levels occurred after the HP/HF meal. When examining each macronutrient separately, protein is reported to have a protective role in hypoglycemia, and fat reduces the glycemic excursion, possibly due to delaying gastric emptying. Regarding insulin adjustments, it is stated that dietary fat increases insulin requirements. Additional insulin boluses should be administered in the form of extended boluses or split boluses, while those using insulin pumps have the advantage of using dual-wave or square-wave boluses [27].

Limited studies have focused on optimizing insulin delivery strategies to reduce the glycemic effects of high-fat and high-protein meals. They evaluated variations in bolus type, timing, and split dosing to achieve improved postprandial glucose control without increasing hypoglycemia risk. In children using insulin pumps, Piechowiak et al. studied the effect of HP/LP on postprandial glucose after normal and dual-wave insulin bolus. The significant difference was reported at 3 h postprandially, with glucose levels being lower when the dual-wave bolus was applied. The study concludes that when a mixed meal containing high protein is consumed, additional insulin should be given in the form of a dual-wave bolus, in order to achieve optimal postprandial glucose levels [6]. Lopez et al. compared different insulin combination regimes after an HF/HP meal, reporting that when a meal contains larger quantities of these macronutrients, insulin boluses should be increased, as the standard dose of insulin is unable to cope with the higher glucose levels. Furthermore, it is stated that for extended insulin boluses, an even greater increase in insulin dose should be made so as to prevent delayed hyperglycemia. For children on multiple daily injections, evidence is less robust [9]. Frohock et al. reported that for children and adolescents with type 1 diabetes treated with multiple daily injections, no significant difference in postprandial glucose levels was observed with additional insulin, despite being reported by other similar studies on both pediatric and adult patients. Smith et al. found benefits when providing a significantly increased insulin dose (125% ICR) preprandially for an HFHP breakfast [14]. The results of those studies are summarized in Table 1 and Table 2. An algorithm used by a few small studies for insulin dose calculation is that of fat–protein units (FPU), first described by Pańkowska et al. to quantify the delayed glycemic impact of dietary fat and protein and incorporate it into insulin dosing calculations for pediatric type 1 diabetes patients using insulin pumps [7]. One FPU is defined as the amount of fat and protein (typically 100 kcal from fat or protein) that requires an insulin dose equivalent to that needed for a certain carbohydrate load. It has been implemented in some pump algorithms to account for the delayed glycemic effects of fat and protein. Subsequent studies, including Smith et al. and Al-Balwi et al., demonstrate that while FPUs can improve late postprandial glucose, individual variability and occasional hypoglycemia remain concerns. All studies, however, note the significant need for further research on the topic of protein and fat in meals, as these macronutrients are included in every mixed meal and they tend to alter patients’ glycemic control [8,23].

Evidence based on small studies suggests that the daily intake of fat and protein can influence glucose metrics, but findings are heterogeneous and do not support the idea that higher intake inherently improves overall glycemic control. In a multicenter cohort study, Cherubini et al. found that higher total fat intake, particularly saturated fat, was associated with lower time-in-range (TIR) and greater glucose variability, even after adjusting for age, BMI, and insulin dose. Moderate protein intake (15–20% of total energy) was linked to improved postprandial stability, but it did not translate into lower HbA1c [4]. Similarly, Muntis et al. investigated habitual dietary patterns and CGM metrics in physically active adolescents. Higher protein consumption was associated with better post-exercise glucose stability, but overall HbA1c and daily TIR were not significantly affected [5]. Taken together, these observational data indicate that simply increasing dietary fat and protein does not reliably improve overall glycemic control in pediatric type 1 diabetes mellitus. Instead, careful attention to macronutrient quality, total energy distribution, and individualized insulin adjustment appears to be more critical for optimizing metabolic outcomes in daily life.

### 3.4. Macronutrient Intake in T1DM Comorbidities

Type 1 diabetes mellitus is usually accompanied by certain comorbid conditions. While some, like celiac disease, are more common than others, like obesity, they may require caloric restriction or gluten-free diets. These dietary alterations reduce carbohydrate intake and shift caloric distribution toward higher protein/fat. ISPAD guidelines recommend an individualized nutrition plan for these patients, emphasizing the need to preserve growth and cardiometabolic health [2]. Observational studies report that rates of overweight/obese T1DM children are increasing and affecting insulin dosing [28]. Studies on a gluten-free diet’s (GFD) effect on glycemic control report mixed findings. Some studies report no adverse effect on HbA1c or BMI, while others suggest a GFD may reduce the insulin requirement but can increase postprandial glucose variability, depending on food choices and processed gluten-free products [29,30]. Future research on these comorbidities and the effects of their corresponding dietary strategies on glycemic control needs to be performed.

**Table 1:** Observational/longitudinal studies.

Study (Year)	Sample Size (*n*)	Focus/Exposure	Outcome
Paterson et al. (2019) [10]	-	Protein impact on glycemia	Protein delays glycemic peaks
Abdou et al. (2020) [26]	51	Postprandial glucose after variations in fat/protein	HFHP meals: Prolonged postprandial glucose ↑ Protein linked to ↑ insulin dose
Cherubini et al. (2021) [4]	197	Weighed daily meals correlated to glycemic response	Higher fat intake → ↑ glycemic variability
Sciacca et al. (2022) [28]	–	Obesity in T1DM	High-fat diets ↑ cardiometabolic risk
Krzewska et al. (2022) [29]	10 studies	Gluten-free diet and glycemia	Mixed metabolic response to GFD
Muntis et al. (2023) [5]	114	High protein intake	High protein → better TIR

T1D: type 1 diabetes, CGM: continuous glucose monitoring, GFD: gluten free diet, *↑*: increase, *→*: resulted in.

**Table 2:** Experimental Meal Studies.

Study (Year)	Sample Size (*n*)	Meal Content	Insulin Strategy	Outcome
Piechowiak et al. (2017) [6]	58	Protein ≥ 30 g	Standard vs. adjusted bolus	High protein: Late glycemic rise—insulin adjustment needed
Lopez et al. (2017) [9]	20	HFHP pizza meal	70/30 dual-wave bolus vs. 60/40 split vs. standard bolus	70/30 dual-wave bolus improved 3–5 h glycemic control
van der Hoogt et al. (2017) [25]	22	Varying fat/protein	Manual insulin adjustments based on meal	HF/HP meals → late hyperglycemia
Frohock et al. (2022) [14]	22	HFHP standardized meals	Additional insulin vs. standard bolus	No consistent benefit: high variability

HFHP: high-fat, high-protein, →: resulted in.

### 3.5. Clinical Implications and Future Directions

International guidelines on diabetes management are published and reviewed thoroughly, with a specific interest in the nutritional management of these patients, and, furthermore, are set on increasing awareness of the glycemic effects of dietary fat and protein. All major international clinical guidelines (ISPAD, ADA) state that while taking into consideration the nutritional benefits of a higher protein and fat intake, it does not equate to improved glycemic control. The International Society for Pediatric and Adolescent Diabetes Clinical Practice Consensus Guidelines 2024, in the specific chapter regarding nutritional management in children and adolescents with type 1 diabetes mellitus, clearly recommend a balanced macronutrient distribution, approximately 45–55% of total energy from carbohydrates, 15–20% from protein, and ≤35% from fat (with saturated fat <10%). Modifications of these percentages could be made individually, depending also on possible co-existing conditions; however, high-fat, high-protein, or low-carbohydrate regimens in children and adolescents should be avoided, due to potential risks for dyslipidemia, micronutrient deficiency, and impaired growth [2]. Similarly, the American Diabetes Association (ADA) Standards of Care 2024, in their Children and Adolescents section of clinical guidelines, suggest a balanced age-appropriate macronutrient intake, with no restriction or emphasis on any single macronutrient [31]. From a clinical standpoint, evidence from the studies performed indicates that higher fat or protein meals demand insulin dose adjustments to correct glucose level fluctuations, and potentially, this is the cause of the observed improved glycemic control in some studies, and not the differences in macronutrient consistency. For pediatric practitioners, it is important to educate families in recognizing the delayed glycemic impact of fat and protein and to use extended or dual-wave bolus strategies in insulin pump therapy when appropriate. Ultimately, clinical nutrition counseling in pediatric type 1 diabetes mellitus should focus on promoting balanced dietary patterns, adequate energy for growth, and cardiovascular health, rather than increasing fat or protein with the expectation of better glycemic outcomes. Future research is needed in order to further investigate the glycemic impact of each macronutrient and should aim to compare diets and nutritional plans with moderate or higher protein/fat intakes, using continuous glucose monitoring (CGM) to quantify time-in-range (TIR), glycemic variability, and insulin requirements. Studies must also evaluate whether different macronutrient proportions affect linear growth, bone health, pubertal development, and lipid profiles in children and adolescents. A matter of critical importance is the insulin algorithms that need to be developed that integrate fat and protein content, particularly within hybrid closed-loop systems. Applying this knowledge in the everyday care of these patients is challenged by substantial individual variability in insulin sensitivity, gastrointestinal physiology, and meal composition, as well as the ways that psychosocial factors, family burden, eating behaviors, and the increasing prevalence of ultra-processed foods may influence the feasibility of dietary strategies. Future research should integrate CGM data, psychosocial outcomes, and hybrid closed-loop technology to establish safe and personalized nutrition strategies, whereas ISPAD and ADA updates should incorporate emerging data to refine evidence-based macronutrient targets and address the long-term safety of novel dietary patterns.

### 3.6. Strengths and Limitations

The evidence accumulated in this narrative review includes a heterogeneous mix of small short-term interventional studies, randomized crossover trials, and observational designs, often with limitations in participant size. Most studies focus on glycemic responses to HF/HP meals, while very few investigate habitual dietary patterns, long-term outcomes, or applicability in everyday care. The absence of standardized definitions for HF/HP meals and variability in insulin dosing strategies further complicate comparisons across studies. Therefore, future research should prioritize well-powered, long-term clinical trials and prospective cohort studies to better evaluate the safety, metabolic consequences, and insulin treatment modifications of macronutrient intake in pediatric T1DM.

## 4. Conclusions

The balance between dietary carbohydrate intake and glucose levels with proper insulin treatment has been the essential element of type 1 diabetes mellitus management. In recent years significant breakthroughs have taken place, with continuous glucose monitoring systems, insulin pumps, and hybrid closed-loop systems all working in favor of patients’ convenience and overall glycemic control improvement. Apart from these advancements, novel ideas regarding dietary macronutrient intake have been proposed, with one being higher protein and fat consumption, and the question of whether it improves glycemic control. Current ISPAD guidelines emphasize individualized, balanced nutrition with moderate fat (30–35% of total energy) and protein (15–20%) intake, aligned with general healthy eating patterns. As mentioned above, many studies have researched the role of protein and fat in glycemic excursions, with both macronutrients causing delayed and prolonged postprandial hyperglycemia when compared with carbohydrate-only meals. Insulin adjustments have been used and studied, and an extended insulin bolus or dual-wave insulin boluses seem to be the most appropriate way of preventing these events. However, all studies conclude that higher fat and protein consumption does not improve glycemic control, and some observed data pointing towards that way (better TIR in a few studies) reflect on the insulin adjustments made rather than the macronutrients themselves. No consistent evidence supports the idea that intentionally increasing the daily fat or protein intake improves overall glycemic control. Future research should focus on refining insulin dosing algorithms, so that they integrate macronutrient composition and continuous glucose monitoring (CGM) data, aiming to improve time-in-range and reduce glycemic variability—without compromising nutritional adequacy or quality of life. The Fat–Protein Unit (FPU) concept and related algorithms offer promising frameworks for adjusting insulin dosing in response to meal composition, but their complexity and variability in pediatric populations currently limit routine clinical implementation.

## Data Availability

No new data were created in this narrative review.

## Abbreviations

The following abbreviations are used in this manuscript:

T1DM	Type 1 diabetes mellitus
ISPAD	International Society of Pediatric and Adolescent Diabetes
ADA	American Diabetes Association
CGM	Continuous Glucose Monitoring
GH	Growth Hormone
Glp-1	Glucagon-like peptide 1
IGF-1	Insulin-like Growth Factor 1
LF/LP	Low Fat/Low Protein
HP/HF	High-Protein/High-Fat
FPU	Fat–Protein Unit
ICR	Insulin-to-Carbohydrate Ratio
TIR	Time-In-Range
GFD	Gluten Free Diet
